# Metabolic syndrome markers and risk of hyperglycemia in pregnancy: a cross-sectional cohort study

**DOI:** 10.1038/s41598-020-78099-3

**Published:** 2020-12-03

**Authors:** Joice M. Vernini, Bianca F. Nicolosi, Mariana A. Arantes, Roberto A. Costa, Claudia G. Magalhães, José E. Corrente, Silvana A. M. Lima, Marilza V. Rudge, Iracema M. Calderon

**Affiliations:** 1 Graduate Program in Obstetrics, Gynecology and Mastology, Botucatu Medical School, São Paulo State University/Unesp, São Paulo, Brazil; 2 Department of Gynecology and Obstetrics, Botucatu Medical School, São Paulo State University/Unesp, Botucatu, SP Brazil; 3 Department of Biostatistics, Botucatu Institute of Biosciences, São Paulo State University/Unesp, São Paulo, Brazil; 4 Department of Nursing, Botucatu Medical School, São Paulo State University/Unesp, São Paulo, Brazil

**Keywords:** Risk factors, Biomarkers

## Abstract

The aim was to assess the role of Metabolic Syndrome (MetS) diagnostic markers, recommended by three different guidelines, in the prediction of hyperglycemia in pregnancy. This cross-sectional cohort study included 506 non-diabetic women, with a singleton pregnancy, who underwent a diagnostic test for hyperglycemia at 24–28 weeks. Clinical, anthropometric, and laboratory data were obtained. The relationship between MetS markers and the risk of hyperglycemia was evaluated by backward stepwise logistic regression analysis (OR, 95% CI). The limit of statistical significance was 95% (*p* < 0.05). Triglycerides (TG) ≥ 150 mg/dL, blood pressure (BP) ≥ 130/85 mmHg, fasting glucose (FG) ≥ 100 mg/dL, and waist circumference (WC) > 88 cm were identified as independent risk factors for hyperglycemia in pregnancy. These results might help the selective screening of hyperglycemia in pregnancy.

## Introduction

Metabolic syndrome (MetS) is a clustering of clinical and laboratory abnormalities that include central obesity, insulin resistance, hypertension, hyperglycemia and dyslipidemia (elevated triglycerides and reduced HDL-cholesterol levels). MetS represents a high socioeconomic burden as it significantly increases the risk of cardiovascular disease (CVD), and metabolic disorders such as type 2 diabetes (DM2)^1–3^. In 2008, Bartha et al.^4^ proposed cutoff values for the diagnosis of MetS during pregnancy.

Gestational diabetes mellitus (GDM) is defined as any degree of glucose intolerance diagnosed in the second and third trimesters of pregnancy with different degrees of maternal hyperglycemia^5^. GDM is the most common metabolic disorder of pregnancy, and it shows facets of MetS including obesity, insulin resistance, and dyslipidemia^6^. Additionally, GDM is associated with adverse short- and long-term effects on both the mother and offspring^7,8^, and increased risk for DM2, MetS and CVD^9^.

In milder forms of GDM, which do not fully meet diagnostic criteria, hyperglycemia and adverse effects to the mother and offspring are also present^10^. In the Perinatal Diabetes Research Centre (PDRC), Botucatu Medical School—UNESP (Sao Paulo State University), Brazil, women with a glycemic profile indicative of hyperglycemia and a normal response to glucose tolerance testing have been identified as having Mild Gestational Hyperglycemia (MGH) and offered the same treatment given to those with GDM for over two decades^11,12^. Today, women with MGH account for 17.3% of our cases^13^.

Current diagnostic guidelines distinguish diabetes mellitus during pregnancy that is diagnosed before 20 weeks of pregnancy from GDM, which is identified during the second and third trimesters. Most frequently, universal screening is performed by initially measuring FG during the initial visit for prenatal care (before 20 weeks of pregnancy). When neither diabetes in pregnancy (FG ≥ 126 mg/dL) nor GDM (FG ≥ 92 and < 126 mg/dL) is detected, a 75 g-OGTT is offered at 24–28 weeks^5,7,14–16^. However, the cost-effectiveness of this strategy, as well as its effects on short- or long-term maternal and offspring outcomes remain unknown and evidence supporting its use is still insufficient^17^.

Recognized risk factors for GDM include advanced maternal age, family history of diabetes, history of GDM, history of macrosomia, non-Caucasian race or ethinicity, excessive weight gain or obesity during pregnancy, and smoking (actively or passively). However, as the prevalence of such risk factors among women with GDM is low (at most 50%)^18,19^ and their predictive accuracy is poor, this may lead to unnecessary testing^17,20^.

The association of MetS with maternal hyperglycemia has been documented since the past decade^21^, and MetS has been shown to be more frequent in women with GDM^4,21^. Studies of BMI, waist circumference (WC), and lipid profile components as independent predictors of GDM have yielded conflicting results^22–25^. However, to the best of our knowledge, the relationship between the MetS markers recommended by different sets of guidelines^2–4^ and risk of MGH or GDM remains uninvestigated. Thus, the objective of this study was to assess the role of the MetS diagnostic markers proposed in three different sets of guidelines—International Diabetes Federation (IDF)^2^, National Cholesterol Education Program’s Adult Treatment Panel III (NCEP-ATP III)^3^, and Bartha et al.^4^, in the prediction of hyperglycemia (MGH or GDM) in pregnancy.

## Methods

### Study setting and design

This cross-sectional cohort study, undertaken from March 2014 to December 2016, included women with a singleton pregnancy who underwent a 75 g-Oral Glucose Tolerance Test (75 g-OGTT) and Glucose Profile (GP) testing between 24 and 28 weeks of pregnancy. The Mets components were evaluated in the inclusion of the study and previously to diagnostic tests. Women with a previous diagnosis of type 1 or type 2 DM, as well as those diagnosed with overt diabetes or GDM before 20 weeks of pregnancy were excluded^5^.

The study was conducted in the Botucatu Public Health Network (the primary care) and in the PDCR–Botucatu Medical School/UNESP, Brazil, our referral center (tertiary care). The study protocol was approved by the Human Subject Research Ethics Committee of Botucatu Medical School/Unesp (# 3900-2011; Of. No. 244/11). All methods were performed in accordance with the principles of the Brazilian National Heath Council (Resolution CNS 466/12) in compliance with local/institutional guidelines and regulations in all stages of this study.

### Sample size

The sample size was based on the prevalence of maternal hyperglycemia between 15 and 20%^13,17^, the risk of 4.21 for hyperglycemia when pre-BMI ≥ 25 kg/m^2^^22^, a type 2 error of 20%, and a 95% confidence level. According to the assumed prevalence, the minimum sample size resulted in 258 and 194, respectively. A total of 506 pregnant women were included, 283 with gestational age < 24 and 223 with gestational age ≥ 24 weeks.

### Data collection

At enrollment, all participants were asked to answer a specific structured questionnaire for the collection of epidemiological and clinical data. These included information on prepregnancy weight, used for the calculus of the prepregnancy BMI, weight gain, and family and personal obstetric risk factors for GDM^5,18,26^. Incomplete or missing information was recovered from the participant’s prenatal care chart. Also at enrollment, were collected data on blood pressure, height and current weight, to calculate the gestational BMI, and waist circumference^27^, besides blood collection for the analysis of FG, glycated hemoglobin (HbA1c), basal insulin and complete lipid profile (LDL and HDL-cholesterol, total cholesterol and TG).

### Variables

The maternal characteristics assessed included: self-reported race (white and non-white), age in complete years (categorized as < 19 years, 19–35 years, and > 35 years); number of pregnancies including current (categorized as 1 and ≥ 2 pregnancies), physical activity (No, Yes, < 150 min/week, and ≥ 150 min/week^28^), smoking status (yes); presence of risk factors for DM^18,26^, and gestacional age at enrollment (< 24 weeks, and ≥ 24 weeks).

The risk factors were defined by MetS diagnostic markers, recommended by IDF, NCEP-ATP III, and Bartha et al. guidelines^2–4^, presented follow:

**Table:** 

MetS componentes	IDF^3^	NCEP-ATP III^2^	Bartha et al.^4^
Central obesity: waist circumference (WC; cm) OR pregestational BMI (Kg/m^2^)	Central obesity: WC ≥ 80 OR pregest BMI ≥ 30	> 88	Abdominal obesity, given as WC > 2 S.D. for gestational age in the first half of pregnancy OR pregest BMI > 30
Triglycerides (mg/dL)	≥ 150	≥ 150	≥ 2 S.D. for gestational age
HDL-cholesterol (mg/dL)	< 50	< 50	< 2 S.D. for gestational age
Blood pressure (mmHg)	≥ 130 / ≥ 85	≥ 130 / ≥ 85	≥ 130 / ≥ 85
Fasting glicose (mg/dL)	≥ 100 OR T2DM	≥ 110	≥ 105
MetS criteria	Central obesity plus any two of the four factors	Any three or more factors	Any three of the five factors

### GDM and MGH diagnoses

All pregnant women included in the study underwent 75-g oral glucose tolerance test (75 g-OGTT) and glucose profile (GP) tests between 24 and 28 weeks of pregnancy.

GDM was diagnosed if there was one abnormal value (92, 180 and 153 mg/dL for fasting, one-hour and two-hour postglucose load, respectively), after a 75 g-OGTT^5,7,14–16^.

For MGH diagnosis, a GP and a 75 g-OGTT were performed during a 1-day hospital stay with the woman on a 2840 kcal-diet fractionated in five meals. Plasma glucose was measured every two hours, from 8 AM to 6 PM. The thresholds used were 90 mg/dL for fasting (8 h) and 130 mg/dL for any postprandial level. MGH was confirmed when response to 75 g-OGTT was normal and one GP measure was equal or greater than threshold values^11,12^.

### Follow-up

Non-diabetic women were followed up at their original primary care center. Women with MGH or GDM were followed up at the PDCR–Botucatu Medical School/UNESP, a tertiary center. In both MGH and GDM cases, maternal hyperglycemia control was performed according to the protocol established in our center as recommended by ADA^5,14^.

### Statistical analyses

Statistical analyses were performed using Statistical Analysis System-9.3.

The results expressed in mean (m) and standard deviation (sd) with symmetric distribution were compared by one-way ANOVA followed by the Tukey test (75 g-OGTT and GP tests, and anthropometric measures); the Gamma test followed by the Wald test (asymmetric distribution) were used to compare means values relative to glucose and lipid profiles. Likewise, Chi-square or Exact Fisher tests (if applicable) were used to test association with MetS criteria according to the guidelines evaluated^2–4^. Two by two proportions were compared using a comparison proportion test based on the normal distribution (similar to chi-square test) by two groups (ND, MGH, and GDM).

The logistic regression model (using backward stepwise) was fitted to identify the independent risk factors for maternal hyperglycemia. It was conducted by two different approaches—one considering all 506 pregnant women included, regardless of the gestational age at enrollment, and another based on metabolic phases of pregnancy, that is < 24 and ≥ 24 weeks in the study inclusion. Here, MGH and GDM were assessed as a unique condition—maternal hyperglycemia as the response variable in function of the MetS diagnostic markers^2–4^. The odds ratio (OR) and 95% confidence intervals (95% CI) were calculated for each MetS diagnostic markers and adjusted for all other variables within the respective guideline. The initial model included all variables/MetS markers, and the variables were excluded one by one until reaching the final model, defined by the impossibility to exclude any other variable without significant loss in adjustment. The backward elimination criterion was 5%.

### Ethical considerations

The study protocol was approved by the Human Subject Research Ethics Committee of Botucatu Medical School/Unesp (# 3900-2011; Of. No. 244/11). All subjects signed an informed consent form before inclusion in the present study.

### Consent for publication

All authors approved the current version and agreed to submit for publication.

## Results

According to 75 g-OGTT response, study participants were assigned to three groups: Non-diabetic (ND, normal 75 g-OGTT and GP); MGH (normal 75 g-OGTT + abnormal GP); and GDM (abnormal 75 g-OGTT + abnormal GP).

**Figure 1:**
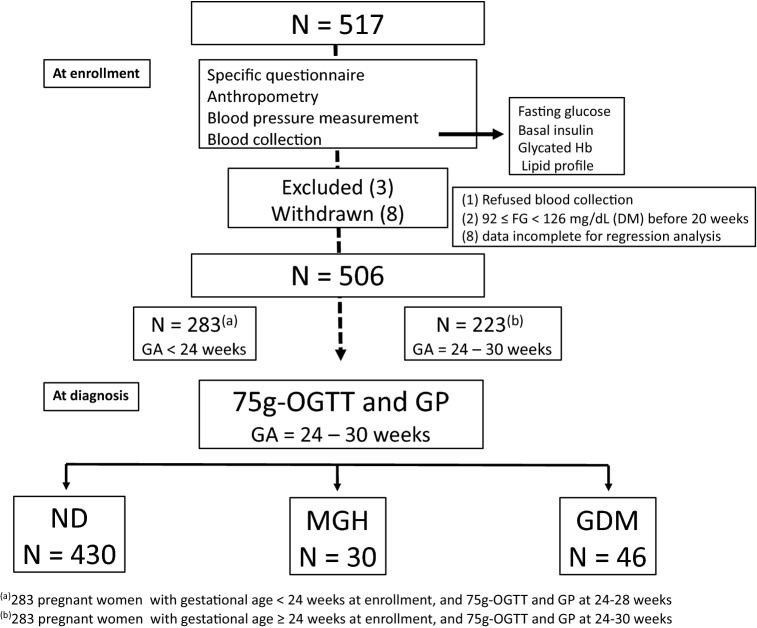
Study flowchart.

The study flowchart (Fig. 1) shows the number of pregnant women included (N = 517), excluded (N = 3), and withdrawn from the study (N = 8), as well as the number of participants in each group—ND (N = 430), MGH (N = 30) and GDM (N = 46). Of the 506 women assessed, 283 (55.9%) were included before 24 weeks and 223 (44.1%) at 24–30 weeks of pregnancy.

The characteristics of the 506 study participants are shown in Table 1. A personal and obstetric history of hypertension (6.5%) and macrossomia (5.1%), and a family history of GDM (58.1%) and hypertension (63.2%) were the most common.

**Table 1:** Study participants’ characteristics.

	N = 506 pregnant women	
	N	%
**Self-reported race**		
White	453	89.5
Non-white	53	10.5
**Age (complete years)**		
< 19	72	14.2
19–35	375	74.1
> 35	59	11.7
**Number of pregnancies**		
1	172	33.60
≥ 2	334	66.40
**Physical activity**		
No	358	70.8
Yes	148	29.2
≥ 150 min/week	51	34.5
< 150 min/week	97	65.5
**Smoking**	125	24.7
**Personal and obstetric history**		
Gestational Diabetes Mellitus	3	0.6
Blood pressure	33	6.5
Polycystic ovary syndrome	4	0.8
Macrossomia	26	5.1
Fetal death	14	2.8
Fetal malformation	10	1.9
**Family history of**		
Diabetes mellitus	294	58.1
Obesity	139	27.5
Hypertension	320	63.2
Cardiovascular disease	182	35.9
Hypercholesterolemia	123	24.4
**Gestational age(GA) at enrollment**		
< 24 weeks	283	55.9
≥ 24 weeks	223	44.1
GA ≤ 12 weeks	154	30.4
GA ≥ 13 and < 19 weeks	81	16.0
GA ≥ 19 and < 24 weeks	48	9.5
GA ≥ 24–30 weeks	223	44.1
GA weeks, mean (sd)	18.9 (7.7)	
GA weeks, median (min; max)	20 (5;30)	

Table 2 shows that the glucose levels in response to diagnostic tests were different among groups, with higher values in the GDM group. Maternal height and the levels of total cholesterol and HDL-c were similar in all groups. The remaining anthropometric and metabolic variables were lower in ND than in the other groups.

**Table 2:** Diagnostic testing results, anthropometric measures, glucose and lipid variables in the study groups ND, MGH and GDM.

	N Total = 506 pregnant women			
	ND N = 430 (mean ± sd)	MGH N = 30 (mean ± sd)	GDM N = 46 (mean ± sd)	*p*-value
75 g-OGTT*				
fasting (mg/dL)	73.0 ± 7.7a	79.2 ± 8.2b	95.9 ± 16.8c	< 0.0001
1 h (mg/dL)	113.1 ± 24.2a	132.1 ± 24.1b	169.2 ± 37.7c	< 0.0001
2 h (mg/dL)	96.2 ± 20.5a	110.1 ± 21.2b	141.2 ± 42.7c	< 0.0001
GP*				
fasting (mg/dL)	74.4 ± 7.1a	87.7 ± 12.8ab	88.2 ± 13.4b	< 0.0001
2 h after breakfast (mg/dL)	85.3 ± 16.7a	101.2 ± 26.5b	113.0 ± 27.7c	< 0.0001
Pre-lunch (mg/dL)	65.9 ± 11.3a	72.2 ± 13.6b	73.8 ± 16.3b	< 0.0001
2 h post-lunch (mg/dL)	92.5 ± 17.2a	121.2 ± 26.7b	109.3 ± 23.9c	< 0.0001
Anthropometric measures*				
Age (years)	26.4 ± 6.49a	28.8 ± 6.8ab	30.6 ± 5.7b	< 0.0001
Height (cm)	1.6 ± 0.06	1.6 ± 0.1	1.6 ± 0.1	0.9982
Pregestational weight (kg)	68.65 ± 17.84a	78.2 ± 22.5b	81.0 ± 23.7b	< 0.0001
Pregestational BMI (kg/m^2^)	26.65 ± 6.66a	30.2 ± 7.3b	31.2 ± 7.7b	< 0.0001
Gestational weight (kg)	72.09 ± 17.58a	81.2 ± 23.7b	85.7 ± 22.7b	< 0.0001
Gestational BMI (kg/m^2^)	27.99 ± 6.49a	31.36 ± 7.6b	33.1 ± 7.2b	< 0.0001
Waist circumference (cm)	95.04 ± 13.6a	101.6 ± 17.4b	108.2 ± 15.5b	< 0.0001
SBP (mm/Hg)	109.87 ± 12.75a	114.0 ± 14.3ab	116.1 ± 13.3b	0.0032
DBP (mm/Hg)	67.39 ± 10.15a	71.3 ± 10.8ab	72.38 ± 10.9b	0.0018
**Glucose and lipid variables**				
Glycated Hb(mg/dL)^#^	4.95 ± 0.44a	5.2 ± 10.6b	5.3 ± 0.4b	< 0.0001
Fasting glucose (mg/dL)*	71.38 ± 8.56a	77.1 ± 12.4b	84.0 ± 13.7c	< 0.0001
Basal Insulina(mg/dL)^#^	8.5 ± 11.2a	16.5 ± 22.1b	12.7 ± 10.1b	< 0.0001
Total cholesterol (mg/dL)*	203.24 ± 45.56	197.7 ± 40.4	212.1 ± 44.5	0.3421
HDL-cholesterol (mg/dL)*	66.43 ± 18.35	63.4 ± 15.7	67.4 ± 13.6	0.6014
Triglycerides (mg/dL)^#^	153.52 ± 66.75a	167.0 ± 82.0ab	198.2 ± 85.7b	0.0003

Values of each specific variable followed by the same letter (a or b or c) are not significantly different (*p* ≥ 0.05).

*ND* non diabetic, *MGH* mildgestationalhyperglycemia, *GDM* diabetes mellitus gestacional, *75 g-OGTT* 75 g oral glucose tolerancetestlicose, *GP* glucose profile, *Pre BMI* body mass index based on pregestational weight, *SBP* systolic blood pressure, *DBP* diastolic blood pressure.

*Anova followed by Tukey test.

^#^Means compared using gamma distribution followed by Wald test.

MetS frequency, as evaluated using NCEP-ATP III^2^ criteria, was 7.0% in ND, 16.7% in MGH, and 21.7% in GDM (*p* = 0.0011).

Regarding the MetS diagnostic markers defined by IDF^3^, the proportion of women with HDL-cholesterol < 50 mg/dL and WC ≥ 80 cm did not differ among groups. The proportion of women with TG ≥ 150 mg/dL, BP ≥ 130/85 mmHg, FG ≥ 100 mg/dL and pregestational BMI ≥ 30 kg/m^2^ was statiscally different among groups with higher values in GDM.

According to NCEP-ATP III^2^ MetS protocol, only the frequency of HDL-cholesterol < 50 mg/dL did not differ among groups, while the proportion of women with TG ≥ 150 mg/dL, BP ≥ 130/85 mmHg, FG ≥ 110 mg/dL and WC > 88 cm was always higher in GDM. Similar results were obtained with the markers recommended by Bartha et al.^4^, adapted for pregnancy (Table 3).

**Table 3:** Association analysis: MetS diagnostic markers^2–4^ and development of MGH or GDM during pregnancy.

	N Total = 506 pregnant women			
	ND N = 430	MGH N = 30	GDM N = 46	*p*-value
**IDF**^3^				
HDL-cholesterol < 50 mg/dL	72 (16.8)	4 (13.3)	3 (6.5)	0.1807
Triglycerides ≥ 150 mg/dL	195 (45.4)a	16 (53.3)ab	35 (76.1)b	0.0003
Blood pressure ≥ 130 / ≥ 85 mmHg	6 (1.4)a	2(6.9)ab	6 (13.3)b	< 0.0001
Fasting glucose ≥ 100 mg/dL	0 (0.0)a	1 (3.3)ab	5 (10.9)b	0.0002
Waist circumference ≥ 80 cm	375 (89.1)	28 (93.3)	45 (100.0)	0.0693
Pregestational BMI ≥ 30 kg/m^2^	107 (24.9)a	13 (43.3)b	20 (43.5)b	0.0039
**NCEP-ATP III**^2^				
HDL-cholesterol < 50 mg/dL	72 (16.8)	4 (13.3)	3 (6.5)	0.1807
Triglycerides ≥ 150 mg/dL	195 (45.4)a	16 (53.3)ab	35 (76.1)b	0.0003
Blood pressure ≥ 130 / ≥ 85 mmHg	6 (1.4)a	2(6.9)ab	6 (13.3)b	< 0.0001
Fasting glucose ≥ 110 mg/dL	0 (0.0)a	1 (3.3)ab	2 (4.3)b	< 0.0001
Waist circumference > 88 cm	286 (66.5)a	24 (80.0)ab	44 (95.7)b	0.0001
**Bartha et al.**^4^				
HDL-cholesterol < 30.50 mg/dL	3 (0.7)	0 (0.0)	0 (0.0)	0.7659
Triglycerides ≥ 299.94 mg/dL	10 (2.3)a	1 (3.33)ab	6 (13.0)b	0.0006
Blood pressure ≥ 130/≥ 85 mmHg	6 (1.4)a	2(6.9)ab	6 (13.3)b	< 0.0001
Fasting glucose ≥ 105 mg/dL	0 (0.0)a	1 (3.3)ab	3 (6.5)b	< 0.0001
Waist circumference > 126.1 cm	5 (1.2)a	1 (3.33)ab	6 (13.3)b	< 0.0001
Pregestational BMI ≥ 30 kg/m^2^	107 (24.8)a	13 (43.3)b	20 (43.5)b	0.0039

Chi-square or Exact Fisher tests (if applicable) to test the association with MetS criteria in each guideline, followed by the comparison proportion test based on the normal distribution (similar to chi-square test) including two groups and two by two proportions.

Values of each specific variable followed by the same letter (a or b) are not significantly different (*p* ≥ 0.05).

*ND* non diabetic, *MGH* mild gestational hyperglycemia, *GDM* diabetes mellitus gestacional, *HDL-cholesterol* high density lipoprotein, *Pre BMI* body mass index based on pregestational weight.

Considering all 506 pregnant women, the logistic regression analysis revealed that TG ≥ 150 mg/dL, BP ≥ 130/85 mmHg, FG ≥ 100 mg/dL and WC > 88 cm are independent risk factors for MGH and GDM, whereas HDL-c < 50 mg/dL and pregestational BMI ≥ 30 kg/m^2^ are not associated with MGH or GDM risk. In contrast, gestational BMI ≥ 30 kg/m^2^, was an independent risk factor for MGH and GDM (OR = 2.796; 95% CI 1.213–6.446) (Table 4).

**Table 4:** Logistic regression analysis: OR and 95%CI of MetS diagnostic markers^2–4^ for predicting MGH or GDMNA.

	N = 506		N = 283 GA < 24 weeks		N = 223 GA ≥ 24 weeks	
	OR	95%CI	OR	95%CI	OR	95%CI
**IDF**^3^						
HDL-cholesterol < 50 mg/dL	0.619	0.265–1.449	1.012	0.385–2.659	0.198	0.024–1.604
Triglycerides ≥ 150 mg/dL	**2.152**	**1.217**–**3.805**	1.030	0.453–2.342	**5.860**	**1.703**–**20.166**
Blood pressure ≥ 130 / ≥ 85 mmHg	**4.853**	**1.553**–**15.172**	2.217	0.394–12.477	**8.420**	**1.325**–**53.510**
Fasting glucose ≥ 100 mg/dL	**26.951**	**2.628**–**276.409**	–	–	**19.845**	**1.845**–**213.493**
Waist circumference ≥ 80 cm	2.245	0.502–10.043	1.995	0.428–9.298	NA*	NA*
Pregestational BMI ≥ 30 kg/m^2^	0.870	0.363–2.087	0.897	0.198–4.070	0.867	0.283–2.652
**NCEP-ATP III**^2^						
HDL-cholesterol < 50 mg/dL	0.545	0.233–1.277	0.962	0.369–2.507	0.173	0.021–1.405
Triglycerides ≥ 150 mg/dL	**2.121**	**1.217**–**3.699**	1.045	0.470–2.325	**5.586**	**1.624**–**19.211**
Blood pressure ≥ 130 / ≥ 85 mmHg	**7.091**	**2.498**–**20.126**	2.618	0.486–14.109	**12.601**	**2.287**–**69–419**
Fasting glucose ≥ 110 mg/dL	**17.199**	**1.376**–**214.963**	–	–	12.601	0.979–162.129
Waist circumference > 88 cm	**3.301**	**1.433**–**7.605**	**2.720**	**1.113**–**6.647**	NA*	NA*
**Bartha et al.**^4^						
HDL-cholesterol < 30.50 mg/dL	–	–	–	–	–	–
Triglycerides ≥ 299.94 mg/dL	**3.978**	**1.313**–**12.051**	**9.891**	**1.191**–**83.167**	2.321	0.592–9.100
Blood pressure ≥ 130 / ≥ 85 mmHg	**4.540**	**1.446**–**14.255**	1.922	0.300–12.337	**11.751**	**1.832**–**75.359**
Fasting glucose ≥ 105 mg/dL	**27.086**	**2.145**–**341.967**	–	–	**20.889**	**1.580**–**276.104**
Waist circumference > 126.12 cm	3.621	0.954–1.755	8.039	0.615–105.043	2.183	0.395–12.053
Pregestational BMI ≥ 30 kg/m^2^	0.652	0.268–1.586	0.779	0.168–3.618	0.648	0.208–2.015
**Gestational BMI ≥ 30 kg/m**^**2**^	**2.796**	**1.213**–**6.446**	2.946	0.677–12.813	2.118	0.731–6.132

*OR* odds ratio, *95% CI* 95% confidence interval, *HDL-cholesterol* high density lipoprotein, *Pregestational BMI* body mass index based on pregestational weight.

*NA = not assessed; no woman with WC < 88 or 80 cm in this gestational age.

According to the gestational age at enrollment, the logistic regression analysis indicated only WC > 88 cm (OR = 2.720; 95% CI 1.113–6.647) as an independent risk factor for MGH and GDM before 24 weeks of pregnancy. At ≥ 24 weeks, TG ≥ 150 mg/dL, BP ≥ 130/85 mmHg and FG ≥ 105 mg/dL (OR = 20.889; 95% CI 1.580–276.104) or ≥ 100 mg/dL (OR = 19.845; 95% CI 1.845–213.493) were identified as independent risk factors for hyperglycemia during pregnancy. In this gestational age, no woman showed WC < 88 or 80 cm (Table 4).

## Discussion

This study evaluated the role of MetS diagnostic markers recommended by different sets of guidelines^2–4^ in the prediction of MGH and GDM risk. In the comparison among groups, the proportion of women with TG ≥ 150 mg/dL, BP ≥ 130/85 mmHg, FG ≥ 100 mg/dL, WC > 88 cm, and pregestational BMI ≥ 30 kg/m^2^ were larger in GDM than in ND group. In the MGH group, these proportions were statistically comparable to those seen in ND and GDM groups. The thresholds HDL-c > 30.5^4^ or 50 mg/dL^2,3^ and CC ≥ 80 cm^3^ did not differentiate the proportion of pregnant women with hyperglycemia from those without. These findings reinforce the proportional relation between MetS and glucose status, as previously reported by our team^21,29^.

Considering all 506 pregnant women, the logistic regression analysis indicate that TG ≥ 150 mg/dL^2,3^, BP ≥ 130/85 mmHg^2,3^, FG ≥ 100 mg/dL^2^ and WC > 88 cm^2^ were identified as independent predictors of MGH or GDM (Table 4). Therefore, according to the physiopathology that points the insulin resistance as the common base to GDM and MetS^3^, these values seem adequate for the diagnosis of MetS during pregnancy. On the other hand, HDL-c < 50^2,3^ or < 30.6 mg/dL^4^, pre-BMI ≥ 30 kg/m^2^^3,4^ and WC ≥ 80 cm^3^ or > 126.1 cm^4^ showed no predictive value to maternal hyperglycemia. However, news values of HDL-c and pregestational BMI should be proposed to MetS criteria in pregnancy.

Before 24 weeks, only WC > 88 cm and TG ≥ 300 mg/dL were independently predictive of risk for MGH or GDM. At 24–30 weeks, TG ≥ 150 mg/dL, BP ≥ 130/85 mmHg and FG ≥ 100 mg/dL were identified as independent predictors of MGH or GDM risk. On the other hand, HDL-c < 50 mg/dL and pregestational BMI ≥ 30 kg/m^2^ showed no predictive value at any gestational age.

Some of the markers evaluated in this study have been previously investigated, but individually and independently from MetS diagnostic guidelines^2–4^. Nonetheless, according to these previous studies, pregestational BMI ≥ 25 kg/m^2^ and WC ≥ 88 cm are predictive of GDM risk^22^; MetS occurrence is directly associated with the level of glucose intolerance^21–29^; obesity (pregestational BMI ≥ 30Kg/m^2^) is associated with MGH or GDM risk, and gestational HbA1c ≥ 6.5%^23^, TG ≥ 137 and 182 mg/dL increase the risk of GDM^30^.

Our findings are consistent with those reported in the literature, but our data regarding FG, BMI and HDL-c are worth of note. The FG values ≥ 100, 105 and 110 mg/dL established in the guidelines for MetS diagnosis^2–4^ are beyond the limit of 92 mg/dL recommended for the diagnosis of GDM^5,7,14–16^. Thus, these results have no clinical application and indicate that FG values should be recalculated and adapted for the diagnosis of MetS during gestation.

In our study, pregestational BMI ≥ 30 kg/m^2^ was not found to be independently predictive of MGH or GDM risk. This was an unexpected finding as it contradicts previous studies^17,20,22,23^. Obesity has been progressively increasing worldwide and, as a result, a larger number of pregnant women has elevated pregestational BMI^3,31^. In our study population, ¼ of ND participants had pregestational BMI ≥ 30 kg/m^2^. Additionally, mean BMI indicated overweight (≥ 25 kg/m^2^) in ND and obesity (≥ 30 kg/m^2^) in MGH and GDM groups. This might have influenced this marker’s ability to predict MGH and GDM and points to the necessity of investigating new threshold limits to prepregnancy BMI.

Among our study participants, HDL-c < 50 mg/dL^2–4^ was not predictive of MGH or GDM. In general, total cholesterol, TG, LDL-c and VLDL-c increase in the second trimester, and are even more greatly increased in the third trimester, whereas HDL-c levels remain unchanged in the second trimester and are decreased in the third trimester. In women with GDM, reduction in lipid measures is even more accentuated^24,25,32^. A recent metanalysis corroborates these results and highlights the progressive increase in TG and significant reduction in HDL-c that occur in the second and third trimesters of pregnancy in women with GDM^33^.

In this study, mean HDL-c levels were higher than the threshold limit of 30.5 and 50 mg/dL proposed with no association with maternal glycemic status. The dynamics of lipid profile during pregnancy and the HDL-c levels observed in our population might explain the inability to predict MGH or GDM, and underscore the need for investigating new HDL-c values to be used in the diagnosis of MetS during pregnancy.

Pregestational BMI and WC have been previously identified as predictors of maternal hyperglycemia risk in Brazil^22,23^ and in other countries^17,20,34–38^. However, the establishment of TG ≥ 150 mg/dL as an independent predictor of risk of MGH and GDM from 24 weeks of pregnancy onward, seem to be contributions unique to this study. The literature shows conflicting results. While some studies do consider TG an independent risk predictor, but only in the first half of pregnancy, others have not associated it with GDM risk^24,30,39^. Nonetheless, the physiological events that take place in the second half of pregnancy—insulin resistance due to the action of the placental hormones, maternal catabolism by rising fetal demands, and a greater increase in TG levels during the third trimester^33,40^ seem to support our findings.

### Study limitations

Although the sample size was enough, the results of the current study should be limited to its setting and population, and future studies in different centers are necessary before clinical application. In addition, gestational age at enrollment, which indicated the timing of MetS markers assessment, ranged from 5 to 30 weeks (total of 26 weeks). Before 24 weeks, gestational age at enrollment ranged from 5 to 23 weeks (total of 19 weeks), and after 24 weeks it ranged from 24 to 30 weeks (total of 7 weeks). Thus, in order to equalize the time periods, the group gestational age < 24 weeks should have been split into 5–12, 13–18, and 19–23 weeks. However, as shown in Table 2, this would reduce the number of samples in each gestational age group and weaken statistical power. In contrast, the novelty in testing the SM markers, defined by three different protocols, in the prediction of the GDM represents the strength of the our study.

### Clinical implications

Our results showed that some MetS markers were identified as independent risk factors for hyperglycemia in pregnancy. So, both in the first and second half of pregnancy, TG ≥ 150 mg/dL, BP ≥ 130/85 mmHg, FG ≥ 100 mg/dL and WC > 88 cm are independent risk factors for MGH and GDM; HDL-c < 50 mg/dL and pregestational BMI ≥ 30 kg/m^2^ are not associated with MGH or GDM risk. In contrast, gestational BMI ≥ 30 kg/m^2^, was an independent risk factor for MGH and GDM.

In the clinical practice, and at any gestational age, TG ≥ 150 mg/dL, BP ≥ 130/85 mmHg, WC > 88 cm, and BMI ≥ 30 kg/m^2^ can be used as screeners associated with the selective diagnostic protocol of MGH or GDM. Ultimately, they would be interpreted as warning signs for hyperglycemia in pregnancy. Considering the cut-off (FG ≥ 92 mg/dL) in the current GDM diagnostic protocol (IADPSG, 2010; ADA, 2011), FG ≥ 100 mg/dL would not useful. The same argument could be done with the results obtained in the first (< 24 weeks) and the second (≥ 24 weeks) half of the pregnancy.

### Research implications

Our results were defined after two different approaches, one based on any gestational age at enrollment, and another according to metabolic phases of pregnancy. Although the sample size was enough for both strategies, the gestational age variation among the subjects (Table 1) may have influenced some results. Moreover, these results are limited to a population of pregnant women with characteristics of their own. Therefore, further studies are needed to (1) reevaluate the threshold limits defined by the MetS diagnostic guidelines; (2) assess the uselfulness of these markers in the prediction of MGH or GDM risk using more restricted gestational age ranges; (3) assess the repeatability of these markers in different populations; (4) to determine the actual role of lipid profile in the physiopathology of hyperglycemia in pregnancy.

Finally, some MetS diagnostic markers recommended by different guidelines can independently predict the risk of MGH and GDM. These findings have important clinical implications as they might help to identify women at risk in the selective screening for hyperglycemia in pregnancy. However, other cut-off points were unable to predict this condition and news studies are necessary to adapt them to metabolic changes of pregnancy.

## Data Availability

The data sets generated and analyzed in the current study may be made available by the corresponding author if requested.
